# From prediction to adaptation: rethinking the epistemic role of inhalation toxicology

**DOI:** 10.3389/ftox.2026.1791543

**Published:** 2026-02-23

**Authors:** Samir Dekali

**Affiliations:** Biomedical Research Institute of the Armed Forces (IRBA), EBR Department, Emerging Technological Risks Unit (U.RTE), Brétigny-sur-Orge, France

**Keywords:** adaptive toxicology, computational modeling, evidence integration, in vitro models, inhalation toxicology, new approach methodologies, uncertainty

## Abstract

Inhalation toxicology has long aimed to predict the health effects of airborne substances before exposure occurs, relying on stable dose-response relationships and well-characterized hazards. This approach becomes increasingly limited when confronted with emerging materials, complex mixtures, and dynamic exposure scenarios, where key mechanisms and variables are not fully known in advance. In this Perspective, we propose reframing inhalation toxicology from a predictive toward an adaptive science, in which experimental and computational systems are designed to rapidly generate and integrate information under conditions of uncertainty. We outline how flexible *in vitro* exposure models, computational dosimetry, and iterative evidence integration can form adaptive frameworks that support learning and updating rather than static prediction. We further discuss the implications of this shift for experimental design, model evaluation, and the handling of uncertainty. This conceptual reframing offers a scientifically grounded approach for maintaining relevance and rigor in inhalation toxicology as exposure landscapes continue to evolve.

## 1 Introduction: inhalation toxicology as a predictive science

Inhalation toxicology has historically been organized around a predictive ambition: to identify hazards, quantify dose-response relationships, and extrapolate findings to human risk. This paradigm has been strongly shaped by the development of standardized toxicity testing strategies and by the emergence of integrated frameworks combining experimental and computational approaches to support regulatory decision-making (Toxicity Testing in the 21st, 2025; OECD, 2020). In this framework, experimental systems and models are primarily designed to generate predictive information for predefined substances and exposure scenarios.

Such a paradigm has proven highly effective for regulated chemicals and well-defined exposure contexts, particularly when exposure conditions, physicochemical properties, and biological targets are relatively stable and well characterized. However, its limitations become apparent when applied to emerging materials, complex mixtures, and dynamic exposure patterns, where relevant variables and mechanisms are incompletely known in advance (Lacroix et al., 2018; Sharma et al., 2023). In these situations, uncertainty is not merely statistical but structural, reflecting gaps in knowledge about exposure, dosimetry, biological interactions, and system-level responses, as well as limitations in translating *in vitro* bioactivity into *in vivo* relevance (Wetmore, 2015; Yoon et al., 2012; El-Masri et al., 2022).

This raises a central question for the discipline: how should inhalation toxicology be structured when prediction is intrinsically limited by the complexity and evolving nature of both exposure scenarios and biological systems?

## 2 The epistemic limits of prediction

Prediction in toxicology relies on the assumption that relatively stable and generalizable relationships can be established between exposure, internal dose, biological perturbation, and adverse outcome. These relationships are formalized through dose-response modeling, mechanistic frameworks such as adverse outcome pathways, and quantitative toxicokinetic models that aim to translate experimental observations into predictive knowledge (Ankley et al., 2010; Krewski et al., 2010).

However, biological systems are inherently complex and adaptive. They exhibit nonlinearity, feedback regulation, context dependence, and emergent behaviors arising from interactions across molecular, cellular, and tissue scales, such that the same perturbation can lead to different outcomes depending on physiological state, prior exposures, or environmental context (Kitano, 2002; Noble, 2012). In inhalation toxicology, this intrinsic biological complexity is compounded by the physicochemical and temporal variability of airborne exposures, including particle size distributions, agglomeration, surface reactivity, chemical transformation during inhalation, and the frequent occurrence of multi-component mixtures rather than single substances (Carpenter et al., 2002; Geiser and Kreyling, 2010).

Therefore, predictive models tend to lose reliability as they are applied outside the specific experimental and exposure domains in which they were developed and calibrated. This limitation does not reflect a failure of modeling, but rather a structural epistemic constraint: in complex biological systems, model parameters are often poorly identifiable and predictive power is necessarily local, limiting robust generalization across contexts and scales (Gutenkunst et al., 2007; Walker et al., 2003).

## 3 Adaptation as a scientific strategy

An adaptive scientific strategy does not replace prediction but complements it by enabling experimental and computational systems to respond to new data, new substances, and evolving exposure scenarios. Rather than assuming that all relevant conditions can be fully defined *a priori*, adaptation emphasizes iterative refinement of methods, updating of models, and rapid integration of new evidence to reduce uncertainty and expand applicability (Sewell et al., 2024).

In inhalation toxicology, adaptation refers to the capacity of experimental and computational systems to incorporate new substances and exposure scenarios without complete redesign, generate informative data under uncertainty, and update conceptual and quantitative models as evidence accumulates. This includes the development and use of flexible *in vitro* platforms, modular exposure designs, and computational frameworks that can be recalibrated or extended as new properties or mechanisms are discovered (Thorne et al., 2024). In operational terms, an adaptive scientific system can be characterized by several key features: iterative updating of experimental protocols and models as new data emerge; explicit identification and tracking of uncertainty; data-driven refinement of endpoints and hypotheses; modular experimental designs that can be reconfigured without complete redevelopment; and predefined decision points that allow reorientation of testing strategies. For example, in a traditional inhalation toxicology framework, a fixed exposure protocol and a limited set of predefined endpoints are selected prior to testing, with model validation performed once against a reference dataset. In contrast, an adaptive workflow may begin with a flexible air-liquid interface exposure system combined with exploratory high-content endpoints, followed by iterative refinement of exposure conditions, endpoints, and computational models as new mechanistic or dosimetric insights are obtained.

Recent developments in the toxicology field illustrate how adaptive strategies can be integrated into test method portfolios. For example, flexible computational toxicology models and integrated in vitro-in silico pipelines allow iterative learning and refinement based on accumulating data streams, rather than fixed, on-off predictions limited to predefined chemical domains (Sewell et al., 2024; Thorne et al., 2024).

This reframing shifts the emphasis from optimal pre-specification toward responsiveness and robustness: the value of a scientific system is defined not only by its performance under a narrow set of conditions, but by its ability to remain informative and adjust as knowledge and exposure contexts evolve.

## 4 New approach methodologies (NAMs) as adaptive scientific systems

New approach methodologies (NAMs) are often presented as alternative testing tools intended to augment or replace traditional animal studies. However, their deeper contribution lies in their flexibility and modularity, enabling experimental and computational systems to adjust as new data and new questions arise. Modern high-throughput screening and integrated *in vitro* approaches produce large, multidimensional datasets that can be explored iteratively to refine hypotheses and guide subsequent testing strategies (Lynch et al., 2024).

In inhalation toxicology, *in vitro* airway and lung models exposed at the air-liquid interface can be adapted to different classes of substances, mixtures, particle types, and exposure dynamics without requiring complete redesign of the entire system, supporting flexible experimental planning across diverse scenarios (Lacroix et al., 2018). For example, recent work using a human lung air-liquid interface co-culture model has shown that repeated exposure to realistic pollutant mixtures can elicit unconventional epithelial-fibroblast signaling responses that are not captured by standard cytotoxicity or inflammatory endpoints, illustrating how flexible NAM platforms can reveal emergent mechanisms under complex exposure conditions (Fredoc-Louison et al., 2025).

At the same time, computational dosimetry, quantitative in vitro-to-in vivo extrapolation, and other *in silico* tools can be recalibrated as new data and mechanistic insights become available, allowing these models to remain informative beyond their initial calibration domain. Such recalibration is a practical manifestation of iterative refinement driven by expanding datasets and improved biological understanding.

High-throughput and high-content approaches further contribute to this adaptive capacity by enabling simultaneous measurement of multiple endpoints in a scalable manner, which facilitates both hypothesis generation and refinement rather than only narrow hypothesis testing. For example, high-throughput methodologies have been successfully applied to generate comprehensive toxicity profiles of thousands of chemicals, illustrating how flexible data generation underpins iterative toxicological assessment (Lynch et al., 2024).

Viewed collectively, these experimental and computational components form an adaptive scientific system capable of navigating uncertainty, accommodating novelty, and integrating new evidence, rather than functioning as a static pipeline limited to predefined and stable conditions.

## 5 Implications for experimental design

Adaptive toxicology requires a shift in experimental philosophy. Rather than optimizing each individual experiment for maximal realism or maximal predictive performance in isolation, priority is given to the design of experimental systems that support comparability, reproducibility, and flexibility across time, laboratories, and exposure scenarios.

In this perspective, standardization is not pursued to enforce uniformity, but to ensure that data generated in different contexts can be meaningfully compared and integrated. Reproducibility across laboratories becomes a central criterion of scientific quality, not only to confirm specific findings, but to ensure that experimental systems remain reliable as they are adapted and reconfigured for new substances, mixtures, or exposure conditions (OECD, 2018; Hartung et al., 2013).

Equally important is the ability to rapidly adjust protocols, exposure conditions, and endpoints without compromising data integrity. This capacity for reconfiguration allows experimental systems to remain informative under changing scientific questions and evolving uncertainties. In this sense, adaptive toxicology does not reduce scientific rigor but redefines it: rigor is expressed less through adherence to fixed experimental forms, and more through reliability, transparency, and interpretability of results as experimental conditions and knowledge evolve.

## 6 Model validation and uncertainty

Traditional model validation in toxicology primarily focuses on the degree of agreement between model predictions and reference or experimental data under specific conditions. While this remains essential, adaptive contexts require a broader view of validation that also considers how models behave when extrapolated beyond their original calibration domain, how sensitive they are to underlying assumptions and parameter choices, and how stable their outputs remain under iterative updating as new data become available.

From this perspective, uncertainty is no longer treated as a residual error to be minimized or ignored, but as an explicit object of analysis that informs both model interpretation and decision-making. Characterizing sources of uncertainty, quantifying their impact on model outputs, and communicating them transparently become integral components of model evaluation, particularly when models are used to support decisions under incomplete knowledge and changing exposure scenarios (Gutenkunst et al., 2007; Walker et al., 2003). Importantly, continuous model validation and incremental calibration are already established practices in computational toxicology and systems biology, where models are routinely updated as new experimental, mechanistic, or exposure data become available. In such contexts, validation is not treated as a one-time comparison against a fixed reference dataset, but as an iterative process involving sensitivity analysis, reassessment of parameter identifiability, and progressive refinement of uncertainty bounds as evidence accumulates (Gutenkunst et al., 2007; Walker et al., 2003). Similar approaches have been applied in exposure and toxicokinetic modeling to support decision-making under uncertainty, illustrating that adaptive validation frameworks are both feasible and scientifically robust rather than merely aspirational.

In adaptive toxicology, validation therefore becomes a continuous process rather than a one-time event. Models are not simply accepted or rejected, but are progressively refined, challenged, and re-evaluated as new evidence accumulates, allowing their domain of applicability, limitations, and reliability to be better understood over time.

## 7 Evidence integration in adaptive frameworks

Adaptive inhalation toxicology requires the structured integration of heterogeneous sources of evidence, including *in vitro* experimental results, computational model outputs, exposure and dosimetry metrics, and mechanistic knowledge. No single data stream is sufficient on its own; scientific insight emerges from their combination, comparison, and mutual constraint within coherent analytical frameworks.

Weight-of-evidence approaches, Bayesian updating, and adverse outcome pathway frameworks provide conceptual tools for organizing and integrating such diverse information. However, in adaptive contexts, these tools must be implemented in ways that support iterative learning rather than static conclusions. Evidence is not accumulated once to reach a final judgment, but is progressively integrated, reweighted, and reinterpreted as new data become available and as models and hypotheses evolve (OECD, 2020; Committee et al., 2017). From a regulatory perspective, adaptive methodologies need not conflict with existing testing and assessment structures. Rather, they can be accommodated through modular validation strategies, tiered integration of evidence, and transparent documentation of model updates and decision points. Frameworks such as Integrated Approaches to Testing and Assessment (IATA) and established weight-of-evidence guidance already provide mechanisms for combining heterogeneous data streams while preserving consistency and traceability in risk assessment. Within such structures, adaptive updating represents a controlled and documented evolution of evidence, rather than methodological instability. Data-driven approaches, including machine learning, can support this adaptive process by facilitating the integration and exploration of high-dimensional and heterogeneous datasets. However, their outputs remain constrained by the quality, scope, and structure of the underlying data, and must therefore be interpreted within the same iterative, transparent, and uncertainty-aware frameworks as other models.

In this perspective, evidence integration becomes a dynamic process that supports exploration, refinement, and prioritization under uncertainty. It enables the scientific system to remain responsive to new findings, to revise earlier interpretations when necessary, and to maintain coherence as the scope of knowledge expands.

## 8 Discussion and conclusion

The future of inhalation toxicology will likely be shaped less by the refinement of individual predictive models than by the development of scientific systems capable of learning, updating, and remaining informative under conditions of uncertainty.

Reframing inhalation toxicology as an adaptive science does not weaken its scientific foundations; on the contrary, it strengthens them by aligning experimental and computational practices with the epistemic characteristics of complex biological systems and evolving exposure landscapes. Prediction remains an important component of this enterprise, but it is embedded within a broader process of iterative evidence generation, model refinement, and uncertainty characterization.

Several elements of adaptive inhalation toxicology are already being implemented in current research practice and, in some cases, within regulatory pilot initiatives. These include flexible *in vitro* exposure systems such as air-liquid interface models, iterative exposure-response analyses, and hybrid workflows combining New Approach Methodologies with computational dosimetry and toxicokinetic modeling. Other components of the adaptive framework proposed here - such as fully dynamic validation schemes, formalized adaptive decision rules, and comprehensive regulatory integration of continuously updating models - remain prospective and represent important directions for future methodological development. Distinguishing between these levels of maturity helps clarify both the feasibility and the forward-looking nature of adaptive toxicology.

In this perspective, rigor is no longer defined primarily by adherence to fixed protocols or static validation criteria, but by the capacity of scientific systems to remain reliable, interpretable, and transparent as they are extended to new substances, mixtures, and exposure scenarios. Adaptive frameworks thus offer a way to sustain both scientific credibility and regulatory relevance in a context where novelty and complexity are no longer exceptional, but structural.

Inhalation toxicology therefore evolves not away from prediction, but beyond it - toward a discipline oriented as much toward learning and adaptation as toward forecasting specific outcomes.

## Data Availability

The original contributions presented in the study are included in the article/supplementary material, further inquiries can be directed to the corresponding author.
